# Comparison of fluid balance and hemodynamic and metabolic effects of sodium lactate versus sodium bicarbonate versus 0.9% NaCl in porcine endotoxic shock: a randomized, open-label, controlled study

**DOI:** 10.1186/s13054-017-1694-1

**Published:** 2017-05-19

**Authors:** Thibault Duburcq, Arthur Durand, Anne-Frédérique Dessein, Joseph Vamecq, Jean-Claude Vienne, Dries Dobbelaere, Karine Mention, Claire Douillard, Patrice Maboudou, Valery Gmyr, François Pattou, Mercé Jourdain, Fabienne Tamion, Julien Poissy, Daniel Mathieu, Raphaël Favory

**Affiliations:** 1 CHU Lille, Centre de Réanimation, F-59000 Lille, France; 2 Univ Lille, INSERM U1190 Translational Research for Diabetes and European Genomic Institute for Diabetes, F-59000 Lille, France; 3 CHU Lille, Centre de Biologie Pathologie, F-59000 Lille, France; 4 CHU Lille, Reference Center for Inherited Metabolic Diseases in Child and Adulthood, F-59000 Lille, France; 5 Medical Intensive Care Unit, Rouen University Hospital, Rouen, France; 6 LIRIC Inserm U995 Glycation: from inflammation to aging, F-59000 Lille, France

**Keywords:** Septic shock, Lactate infusion, Fluid balance, Metabolism, Organ failure, Microcirculation

## Abstract

**Background:**

Sodium lactate has been shown to improve hemodynamics and avoid fluid overload. The objective of this study was to confirm a beneficial effect on fluid balance with sodium lactate infusion and to specify whether the advantage of lactate is related to a negative chloride balance, its particular metabolism, or simply its energy load.

**Methods:**

This was an interventional, randomized, open-label, controlled experimental study. Fifteen female “large white” pigs (2 months old) were challenged with intravenous infusion of *Escherichia coli* endotoxin. Three groups of five animals were randomly assigned to receive different fluids: a treatment group received sodium lactate 11.2% (SL group); an isotonic control group received 0.9% NaCl (NC group); and a hypertonic control group, with the same amount of osmoles and sodium as the SL group, received sodium bicarbonate 8.4% (SB group). In order to provide the same energy load in the three groups, control groups were perfused with an equivalent energy supply. Statistical analysis was performed with non-parametric tests and the Dunn correction for multiple comparisons at *p* < 0.05.

**Results:**

Fluid and chloride balance, hemodynamics, oxygenation markers, and microcirculatory parameters were measured over a 5-h period. Cumulative fluid balance was significantly lower in the SL group (550 (415–800) mL; median (interquartile range)) compared to the NC group (1100 (920–1640) mL, *p* = 0.01) and the SB group (935 (790–1220) mL, *p* = 0.03). Hemodynamics, cardiac efficiency, and microcirculation were significantly enhanced in the SL group, resulting in a significant improvement in oxygen delivery (SL group 417 (305–565) mL/min/m^2^ at 300 min versus the NC (207 (119–272) mL/min/m^2^, *p* = 0.01) and the SB (278, (211–315) mL/min/m^2^, *p* = 0.03) groups). Oxygenation markers (arterial oxygen partial pressure (PaO_2_)/inspired oxygen fraction (FiO_2_), mixed venous oxygen saturation (SvO_2_), and venoarterial carbon dioxide tension difference (Pv-aCO_2_) were enhanced with sodium lactate infusion. Chloride balance was equivalent in both hypertonic groups and significantly reduced compared to the NC group.

**Conclusion:**

Sodium lactate infusion improves fluid balance and hemodynamics. The advantage of lactate does not seem to be explained by its energy load or by the induced negative chloride balance with subsequent water movements.

**Electronic supplementary material:**

The online version of this article (doi:10.1186/s13054-017-1694-1) contains supplementary material, which is available to authorized users.

## Background

Sepsis, considered today as a syndrome of physiologic, pathologic, and biochemical abnormalities induced by infection [1], is a major public health concern responsible for considerable morbidity and mortality [2]. Sepsis is frequently associated with a deficit in effective blood volume. Large amounts of intravenous fluid are commonly used to increase cardiac output and improve peripheral blood flow [3]. However, the clinical determination of intravascular volume can be extremely difficult, and dosing intravenous fluid during resuscitation of shock remains largely empirical [4]. In daily practice, the assessment of individual thresholds in order to avoid hypovolemia or deleterious fluid overload remains a challenge. Whereas under-resuscitation results in inadequate organ perfusion, accumulating data suggest a relationship between over-resuscitation with positive fluid balance and mortality [5–8], but whether this represents a simple association or a cause-and-effect relationship remains unclear [9]. Liberal versus restrictive fluid management has been challenged by recent evidence, and the ideal approach appears to be an adequate initial resuscitation of patients in shock followed by a later more conservative fluid management [10]. This current strategy is often difficult to achieve with standard crystalloid solutions. In order to avoid excessive fluid, the concept of small volume resuscitation with hypertonic solutions has been developed. Low volumes of hypertonic saline fluids may have beneficial effects on the global circulation and the cardiac function that exceed simple intravascular volume expansion [11]. However, the presence of supraphysiological concentrations of chloride may increase the incidence of acute kidney injury and the use of renal replacement therapy [12]. Hence, in an attempt to avoid the detrimental effects of chloride anion, the use of metabolized anions such as lactate could be more suitable. The use of lactate as a resuscitation fluid-based energetic substrate is an interesting alternative because this anion is well metabolized [13] even in poor hemodynamic conditions [14]. Moreover, in several conditions, sodium lactate improves hemodynamics and, simultaneously, avoids fluid overload [15–18]. We previously observed a better macrocirculatory and microcirculatory preservation together with a more negative fluid balance in a swine endotoxic model [19]. To date, three theoretical hypotheses may explain the preservation of a fluid balance with sodium lactate: first, an improvement in organ function by caloric intake; second, an improvement in organ function by a specific metabolic pathway; and third, a chloride balance with subsequent water movements.

Thus, we decided to conduct new experiments to confirm a beneficial effect on fluid balance and to determine what mechanism would be predominant in inflammatory conditions. As suggested by Fontaine et al. [20] in an editorial, we gave similar amounts of caloric intake to test the hypothesis of caloric effect in our previous control groups. Moreover, we decided to take urinary samples (with chloride dosage) at each timing of the experiment scheme to better investigate the evolution of the chloride balance between groups throughout the experiment.

## Methods

We performed a randomized, open-label, controlled experimental study approved by the Institutional Review Board for Animal Research (protocol CEEA n°132012). Care and handling of the animals were in accordance with National Institutes of Health guidelines. We used here the same model already described in our previous work [19]. Detailed methods are available in Additional file 1.

### Study design

Fifteen female “large white” pigs (2 months old) were used in this study. The study was carried out as depicted in Additional file 2. Measurements were taken over a 5-h period: two times at baseline (before (T) and after (T0) the stabilization period), and at 30 (T30), 60 (T60), 120 (T120), 210 (T210), and 300 (T300) min. All animals were administered 5 μg/kg/min *Escherichia coli* lipopolysaccharide (LPS; serotype 055:B5; Sigma Chemical Co., St. Louis, MO, USA). The only resuscitation endpoint was mean arterial pressure (MAP). If MAP fell below 65 mmHg, 2.5 mL/kg infusion of NaCl 0.9% was given as rescue therapy every 15 min. Bolus infusions were performed to maintain MAP above 65 mmHg as recommended by the Sepsis Surviving Campaign [3]. At the end of the study period, all animals were sacrificed with T61 administration (T61, 0.3 mL/kg of body weight; Intervet International GmbH, Köln, Germany).

We studied three groups receiving 450 mL (from T30 to T300) of different fluids as follows: a treatment group (*n* = 5) receiving 11.2% hypertonic sodium lactate AP-HP® (AGEPS, Paris, France) (the SL group) containing 90 g lactate and 23 g sodium per liter; and two control groups: one isotonic control group (*n* = 5) receiving 0.9% NaCl (the NC group), and one hypertonic control group (*n* = 5) receiving 8.4% hypertonic sodium bicarbonate (the SB group) containing 61 g bicarbonate and 23 g sodium per liter. Sodium bicarbonate provided the same amount of sodium and osmoles (2000 mosm/L) and the same alkalizing effect as sodium lactate [19]. The SL group received 450 mL hypertonic sodium lactate, equivalent to 40.5 g lactate (3.61 kcal/g). Conversely to our previous study [19], the NC and SB groups received 39 g glucose (3.75 kcal/g) as 780 mL 5% glucose solution (Baxter SAS, Guyancourt, France) from T30 to T300 in order to infuse an equivalent energy supply as in the SL group. Finally, in order to maintain the same fluid intake in the three groups, the SL group received 780 mL sterile water for injection (Baxter SAS, Guyancourt, France) in place of the 5% glucose solution from T30 to T300.

The primary endpoint was to confirm a beneficial effect on fluid balance with sodium lactate infusion compared to control fluids (saline and sodium bicarbonate) perfused with an equivalent energy supply. Secondary endpoints were hemodynamics, oxygenation markers, and microcirculatory parameters.

### Macrocirculatory parameters and fluid balance

Catheters were inserted in to the pulmonary artery via the right external jugular vein (Swan-Ganz; Baxter 110 H 7.5 F; Baxter Edwards Critical Care, Irvine, CA, USA) and in to the right carotid artery for continuous blood pressure monitoring and blood sampling. The heart rate, systemic, and pulmonary arterial pressures were continuously monitored (90308 PC Express Portable Monitor, Spacelabs, USA) as well as cardiac output and mixed venous oxygen saturation (SvO_2_; Vigilance monitor; Baxter Edwards, Irvine, CA, USA). Using standard formulae, we computed global oxygen delivery (DO_2_), global oxygen consumption (VO_2_), oxygen extraction ratio (OER), cardiac index (CI; L/min/m^2^), systolic index (SI; mL/b/m^2^), and systemic vascular resistance (SVR; dynes/s/cm^5^). To monitor urine output, a suprapubic urinary catheter was inserted by minilaparotomy. Urine output was measured at T0, T60, T120, T210, and T300. Fluid balance from T0 to T300 was determined by calculating the difference between the cumulative amounts of fluids administered minus the cumulative amounts of fluids collected in the urine bag.

### Microcirculatory parameters

Skin microvascular blood flow was measured continuously using a laser Doppler flowmeter probe and device (Periflux® PF407; Perimed, Jiirfalla, Sweden). The fiberoptic probe was applied to the left hind paw of the animals. Skin blood flow was measured at rest and during reactive hyperemia. Reactive hyperemia was further analyzed (Perisoft® 2.5 software) according to its duration and initial reactive hyperemia uphill slope. Basal tissue oxygen saturation (StO_2_) was evaluated using a near-infrared spectrometer (NIRS; InSpectra® 850 model, Hutchinson Technology, Hutchinson, Minnesota, USA), with a probe attached on the pectoral skin area [21]. We were not able to perform a vascular occlusion test because the probe was too large and could not be applied to a paw. Images of the sublingual and rectal microcirculation were obtained with SDF videomicroscopy (Microscan®; Microvision Medical, Amsterdam, The Netherlands). Assessment of microcirculatory parameters of convective oxygen transport (microvascular flow index (MFI)) was made in parallel [22].

### Biological methods

Systemic arterial and venous blood samples from carotid and pulmonary arteries were obtained simultaneously. Arterial and venous blood gas tensions and lactate levels were measured in an acid-base analyzer (ABL-800, Radiometer, Copenhagen, Denmark) at each time except T30. Blood and urinary electrolyte concentrations and osmolality, urea, creatinine, total protein, and bilirubin levels (Cobas® 8000modular analyser, Roche Diagnostics, Switzerland) were measured at the same time. Capillary glucose (Accu-Chek PERFORMA®, Roche Diagnostics SAS, Meylan, France) and insulin levels (Architect® i2000SR Immunoassay analyser, Abbott diagnostics, Abbott Park, Illinois, USA), lactate, pyruvate, beta-hydroxy-butyrate and acetoacetate, glycerol, and non-esterified fatty acids (NEFA) were also analyzed at the same time.

### Data analysis

We considered that the sample size of five animals per group would be sufficient to show a statistical difference in fluid balance, if any, based on our previous work [19]. Statistical analysis was performed with GraphPad Prism 6 software (San Diego, California, USA). As the distribution was not normal (Shapiro-Wilk test), quantitative data were expressed as median and interquartile range. Considering the differences between groups for some parameters at baseline, values were expressed as a percentage of the first value. For multiple inter-group testing we used the Kruskal-Wallis test with Dunn’s multiple comparisons test and Mann-Whitney *U* test. Intra-group comparisons were realized by Friedman test with Dunn’s multiple comparisons test. The two-tailed significance level was set at *p* < 0.05.

## Results

Median weight was similar in the three groups of animals: 22.5 (18.25–23.75) kg in the NC group, 23 (21.75–23.5) kg in the SB group, and 23 (20.5–24) kg in the SL group. No animals died during the 5-h experiments.

### Fluid balance

**Fig. 1:**
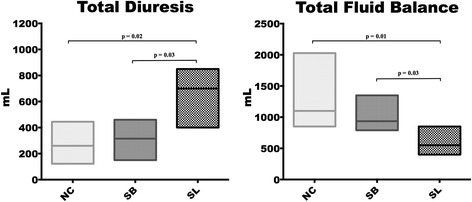
Total diuresis and total fluid balance. Results are expressed as median with interquartile ranges. *NC* isotonic control group receiving NaCl (*n* = 5), *SB* hypertonic control group receiving sodium bicarbonate (*n* = 5), *SL* treatment group receiving hypertonic sodium lactate (*n* = 5)

Urine output and fluid balance at baseline were similar among the groups. At 210 and 300 min, urine output was significantly higher in the SL group compared to the NC and SB groups, resulting in a significantly lower fluid balance (Fig. 1).

### Macrocirculatory and oxygenation parameters

**Fig. 2:**
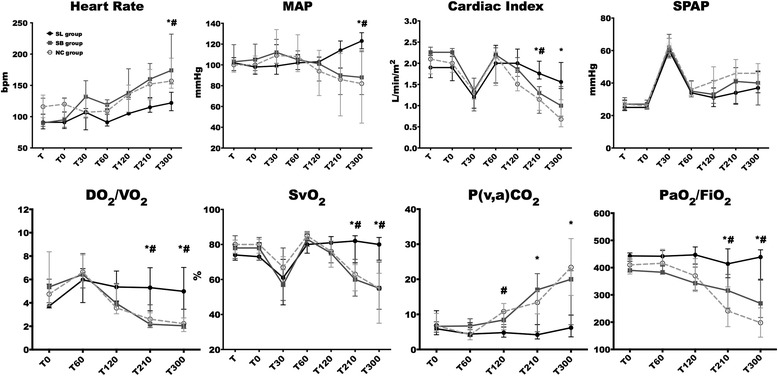
Changes in heart rate, mean arterial pressure (*MAP*), cardiac index, systolic pulmonary arterial pressure (*SPAP*), oxygen delivery/oxygen consumption (*DO*_*2*_ */VO*_*2*_) ratio, mixed venous oxygen saturation (*SvO*_*2*_), venoarterial CO_2_ tension difference (*Pv-aCO*_*2*_), and inspired oxygen fraction ratio (*PaO*_*2*_ */FiO*_*2*_). *p < 0.05, NC versus SL; ^#^ *p* < 0.05, SB versus SL. *NC* isotonic control group receiving NaCl (*n* = 5), *SB* hypertonic control group receiving sodium bicarbonate (*n* = 5), *SL* treatment group receiving hypertonic sodium lactate (*n* = 5)

No changes in the studied variables were observed during the stabilization period. Endotoxin infusion resulted in similar and usual changes until 60 min as previously described [23, 24]. A better hemodynamic stability, with statistically higher MAP and cardiac index at 300 min, was observed in the SL group (Fig. 2). Throughout the study, four (two in the NC group, two in the SB group, and none in the SL group) animals required 0.9% NaCl boluses to maintain MAP ≥65 mmHg according to the study protocol. Right atrial pressure and SVR changes were similar in the three groups without any significant difference (Additional file 3). DO_2_ decreased in the control groups between 60 and 300 min, while it remained stable in the SL group. At 300 min, compared to the SL group (417 (305–565) mL/min/m^2^), DO_2_ was significantly lower in the NC (207 (119–272) mL/min/m^2^; *p* = 0.01) and SB (278 (211–315) mL/min/m^2^; *p* = 0.03) groups. At the same time, VO_2_ remained stable in the three groups without a significant difference resulting in increased OER only in the control groups (Additional file 3). In parallel, SvO_2_ decreased from baseline to 300 min by 36% in the NC group and 30% in the SB group, while it remained stable in the SL group (Fig. 2). The venoarterial CO_2_ tension difference (Pv-aCO_2_) increased and the arterial oxygen partial pressure and inspired oxygen fraction ratio (PaO_2_/FiO_2_) ratio decreased in the control groups while it remained stable in the SL group (Fig. 2).

### Microcirculatory parameters

**Fig. 3:**
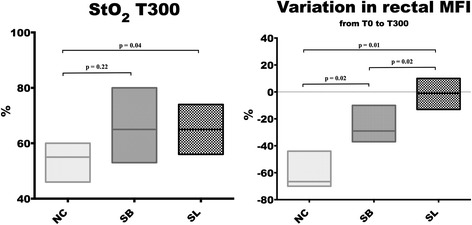
Changes in basal tissue oxygen saturation (*StO*_*2*_) and variation in rectal microvascular flow index (*MFI*) score between 0 min (*T0*) and 300 min (*T300*). Results are expressed as median with interquartile ranges. *NC* isotonic control group receiving NaCl (*n* = 5), *SB* hypertonic control group receiving sodium bicarbonate (*n* = 5), *SL* treatment group receiving hypertonic sodium lactate (*n* = 5)

Skin microvascular blood flow decreased in the three groups. However, rest flow (RF) and peak flow (PF) were significantly more impaired at the end of the study in the NC group compared to the hypertonic groups. No difference was observed between the SL and SB groups (Additional file 4). In the same way, basal StO_2_ decreased significantly only in the NC group and was significantly lower at T300 (55 (48.5–64.5)) compared to the SL (65 (58–71), *p* = 0.04) group. The sublingual MFI score decreased in all groups without any statistical difference between groups. The rectal MFI score decreased significantly in the NC and SB groups while it remained stable in the SL group from T0 to T300 (Fig. 3).

### Biological parameters

As expected, pH, bicarbonate levels, blood osmolality and blood sodium levels were similar in the SB and SL groups, and were significantly higher compared to the NC group (Additional files 5 and 6).

**Fig. 4:**
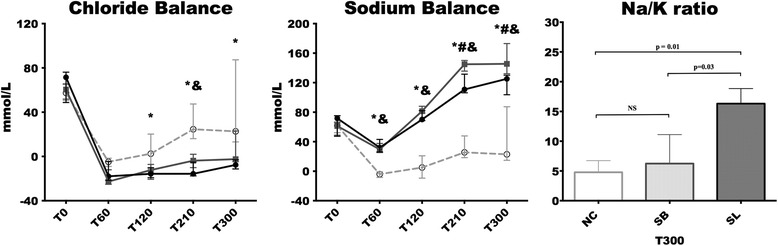
Changes in chloride and sodium balances, and the Na/K ratio at 300 min (*T300*). Results are expressed as median with interquartile ranges. *p < 0.05, NC versus SL; ^#^ *p* < 0.05, SB versus SL; &*p* < 0.05, NC versus SB. *Open circles and dotted line*: *NC* isotonic control group receiving NaCl (*n* = 5); *squares and grey line*: *SB* hypertonic control group receiving sodium bicarbonate (*n* = 5); *closed circles and black line*: *SL* treatment group receiving hypertonic sodium lactate (*n* = 5)

Mean chloride balance from T0 to T300 was significantly lower in the SB (–42.56 (–55.37 to 1.13) mmol/l) and SL (–61.80 (–67.22 to –22.91) mmol/l) groups compared to the NC group (48.29 (24.27–136.1) mmol/l; *p* = 0.01, respectively) without a significant difference between the SB and SL groups. The chloride balance was non-significantly lower in the SL group (–15.68 (–17.54 to –5.12) at T210 and –7.65 (–11.27 to –3.02) at T300) compared to the SB group (–3.84 (–10.10 to 1.95) at T210 and –2.24 (–4.71 to 23) at T300; *p* = 0.09, respectively). Mean sodium balance from T0 to T300 was significantly higher in the hypertonic groups compared to the NC group (*p* = 0.008, respectively) without a significant difference between the SB and SL groups. Nevertheless, due to a significantly higher natriuresis at 210 and 300 min in the SL group compared to the SB group, sodium balance at the same time was significantly lower in the SL group (T210: 110.8 (106.3–131.5); and T300: 125.3 (103.7–130.1) mmol) compared to the SB group (145 (135.7–150.1), *p* = 0.02; and 145.5 (132.1–73), *p* = 0.02) (Fig. 4 and Additional file 6). In parallel to natriuresis, the Na/K ratio was significantly higher in the SL group compared to the NC (*p* = 0.01) and SB (*p* = 0.03) groups at T300 (Fig. 4).

### Metabolic results

**Fig. 5:**
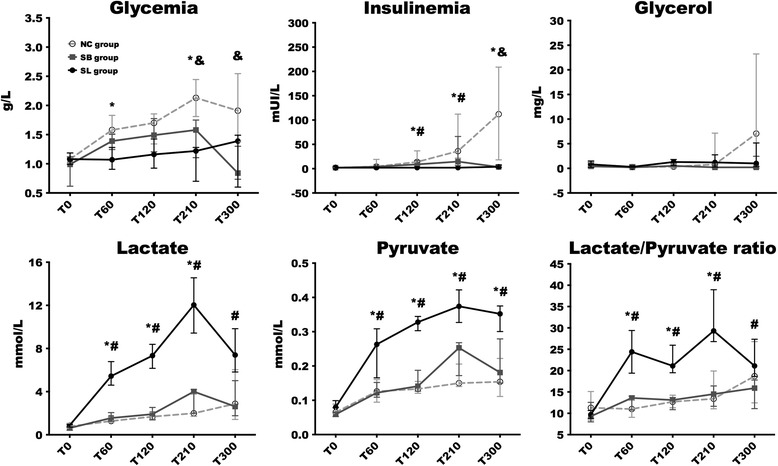
Changes in metabolic parameters. Results are expressed as median with interquartile ranges. *p < 0.05, NC versus SL; ^#^ *p* < 0.05, SB versus SL; &*p* < 0.05, NC versus SB. *NC* isotonic control group receiving NaCl (*n* = 5), *SB* hypertonic control group receiving sodium bicarbonate (*n* = 5), *SL* treatment group receiving hypertonic sodium lactate (*n* = 5)

Glycemia, insulinemia, and glycerol levels increased at the end of the experiment in the NC group while it remained stable in the SB and SL groups (Fig. 5). A similar increase in lactate and pyruvate levels was observed in the NC and SB groups. Lactate and pyruvate levels increased earlier in the SL group and peaked at 210 min (12.03 (9.427–14.55) and 0.374 (0.327–0.422) mmol/L, respectively). Ketone bodies (acetoacetate and beta-hydroxy-butyrate) and non-esterified fatty acid levels remained stable without any significant variation in the three groups (data not shown).

## Discussion

In this study, we report that the infusion of sodium lactate enhances fluid balance, hemodynamics, oxygenation markers, and microcirculatory parameters. Hence, we confirm our previous results [19]; however, this time all the animals received the same amount of calories and we investigated the evolution of the chloride balance between groups throughout the experiment.

The advantage of lactate does not seem to be simply explained by its energy load. In fact, in the present study, all animals received the same caloric load. As a marker of shock severity, only the NC group developed insulin resistance (reflected by the increase in glycemia and insulinemia levels) and inhibition of gluconeogenesis (reflected by the increase in glycerol levels). Otherwise, the present study demonstrates that lactate, if used as an energy substrate, can help to avoid hyperglycemia under conditions of septic shock.

The advantage of lactate could not be explained by the chloride balance. As already described [19], we observed a negative chloride balance following SL infusion, which supported chloride urine excretion coupled to the high sodium urine excretion. In fact, the important amount of urinary sodium excretion creates an imbalance between positive and negative charges. As the urine is poor in protein (positive charges could be neutralized by the increase of negative charges on protein), a net anion efflux must therefore compensate the excess of positive charges in order to maintain electroneutrality. This is achieved in a substantial part by an increase in urinary chloride excretion [16, 25]. Chloride, the principal intracellular inorganic anion, is responsible for intracellular tonicity. In fact, recent data on Na+, K+, and Cl^–^ co-exchange support a significant role of chloride balance on cell volume regulation [26, 27]. Hence, the net efflux of chloride could be accompanied by a net flux of water and could participate in the larger urine output with SL infusion compared to the NC group. However, we observed the same chloride balance in the SL and SB groups. Thus, the hypertonic beneficial effect with sodium/chloride imbalance that may induce a negative fluid balance equivalent in the SL and SB groups could not explain the difference in fluid balance between the hypertonic groups.

The lactate-specific metabolic pathway may have contributed to improve hemodynamics and fluid balance. In fact, the induction of metabolic alkalosis by SL infusion could reflect the lactate oxidation. Moreover, the rapid increase in pyruvate after sodium lactate infusion strongly suggested a conversion of lactate to pyruvate. To our knowledge, this is the first time this last point has been highly suggested by in vivo data. This metabolic support could explain the better cardiac efficiency in the SL group. Indeed, evidence is now accumulating that sodium lactate may serve as a resuscitation fluid-based energetic substrate that is readily oxidizable and provides an important fuel for tissue energetics under stress or energy crisis conditions [28, 29]. It is known that lactate may improve myocardial function during shock [30, 31] and it has been shown that lactate deprivation during shock impairs heart metabolism [32].

In our model, as already described [19], the hemodynamic effects of lactate infusion seem well related to an improvement in cardiac function rather than to changes in vascular tone. SVR was not significantly modified by lactate administration, whereas CI and systolic index were higher. The rectal microcirculation was significantly enhanced in the SL group compared to both control groups. Consequently, the Pv-aCO_2_ gradient remained low in the SL group while it increased significantly in the NC and SB groups. This last parameter argues for a better macro- and microcirculatory status with sodium lactate perfusion. Finally, fluid balance was significantly lower in the SL group compared to the NC and SB groups. The higher volume of urine output in the SL group could be easily explained by better macro- and microcirculatory status compared to the NC group. Nevertheless, a better hemodynamic status could not explain the large difference in fluid balance between the SL and SB groups. In fact, MAP and CI in the SB group were less decreased than in the NC group.

Another explanation for the prevention of fluid overload with sodium lactate could be an improvement in endothelial barrier function, as recently described in pediatric severe Dengue infection [17]. Although the pathogenesis of endotoxic shock is different from septic shock, endothelial cell dysfunction is common in both situations. In fact, porcine endotoxic shock is characterized by a severe vascular systemic capillary leak syndrome, and histologic findings supported the development of a respiratory distress syndrome with marked pulmonary leukocyte sequestration and interstitial edema [33]. In our model, sodium lactate infusion appeared to reduce capillary leakage, particularly in the lung. In fact, sodium lactate infusion preserved the PaO_2_/FiO_2_ ratio compared to the control groups, and the SL group seemed to develop a less-pronounced hypovolemia as reflected by lower tachycardia. As suggested by Somasetia et al. in dengue shock syndrome [17], we hypothesized that endotoxin challenge leads to endothelial cell swelling, which in turn increases endothelium permeability because of changes in the cell shape. We propose that lactate may lead to chloride egress from endothelial cells [34], causing the cell volume to revert back to normal, thus correcting capillary leakage. Finally, it was recently shown that lactate by itself has important anti-inflammatory properties through the activation of a specific membrane receptor that may lead to a reduced inflammatory response and endothelial activation [35].

A recently published study [36] may be viewed as challenging our results. Su et al., in an ovine model of septic shock, have shown that administration of half-molar sodium lactate resulted in an earlier onset of impaired tissue perfusion and a shorter survival time than similar infusions of 3% hypertonic saline. In our view, two main explanations can be drawn. 1) An insufficient dose of lactate; the total amount of lactate infused was only 200 mEq for a 15-h period whereas we perfused 450 mEq for a 4.5-h period (more than seven times more); hence lactate represents less than 10% of the total infused volume. 2) An under-resuscitated group; the only hemodynamic target was to maintain the pulmonary artery occlusion pressure (PAOP) at baseline levels. Consequently, the hypertonic sodium lactate group received 30% less total fluid intake than the hypertonic saline group. Thereby, the smaller fluid intake could explain the earlier impairment of tissue perfusion in this model of distributive shock.

The authors hypothesized that hypertonic sodium lactate-induced metabolic alkalosis is the most likely reason for the early decrease in tissue perfusion. Unfortunately, a control group with the same alkalinizing effect as sodium lactate was not performed to confirm this hypothesis.

Our model presents some limitations. The length of evaluation is short at only 5 h, and the endotoxic model is not a model of hyperdynamic septic shock. However, it reproduces some of the main alterations of inflammatory states, e.g., macrocirculatory and microcirculatory dysfunctions, insulin resistance, and capillary leakage, etc. We chose a MAP target of 65 mmHg, as in humans, which may be criticized. Yet, in a preclinical model of septic shock it seemed pragmatic to us to use a widely accepted MAP target in clinical practice. There were a limited number of animals per group; thus, a limited power in our statistical analyses. However, as in our previous work, we were able to detect statistical differences between groups. Finally, infusion of such a large amount of lactate will modify the serum lactate level and kinetics, and will have to be taken into account in interpreting lactate monitoring during the initial resuscitation.

## Conclusions

In conclusion, sodium lactate infusion improves fluid balance, hemodynamics, oxygenation markers, and microcirculatory parameters in our model of endotoxic shock. The effects of sodium lactate do not seem to be explained by its energy load or its effect on the chloride balance with subsequent water movements. Two mechanisms may be advanced: first, lactate infusion is beneficial as a metabolic supplier better metabolized in poor hemodynamic conditions than glucose; and second, lactate could reduce the inflammatory response and endothelial activation attenuating capillary leakage. Further investigations are warranted to explain the underlying mechanisms and to assess the potential clinical benefits of sodium lactate resuscitation in sepsis.

## Additional files

Additional file 1: Detailed materials and methods. Animal preparation, microcirculatory parameters, biological, and metabolic methods are specifically described. (TXT 5 kb)

Additional file 2: Study design. During preparation period, all animals received 25 mL/kg 0.9% NaCl to prevent hypovolemia. When all preparations were completed, a 30-min period was allowed to stabilize the measured variables. Measurements were taken over a 5-h period. All animals were administered 5 μg/kg/min *Escherichia coli* lipopolysaccharide (LPS) (serotype 055:B5; Sigma Chemical Co., St. Louis, MO, USA). If MAP fell below 65 mmHg, 2.5 mL/kg infusion of NaCl 0.9% was given as rescue therapy every 15 min. We studied three groups receiving 450 mL (from T30 to T300) of different fluids as follows: 11.2% hypertonic sodium lactate AP-HP® (AGEPS, Paris, France) (SL group), 0.9% NaCl (NC group), and 8.4% hypertonic sodium bicarbonate (SB group). In order to inject an equivalent energy supply, 5% glucose solution (Baxter SAS, Guyancourt, France) was perfused in the NC and SB groups. Finally, in order to maintain the same fluid intake in the three groups, the SL group received 780 mL sterile water for injection (Baxter SAS, Guyancourt, France) in place of 5% glucose solution from T30 to T300. SL, Sodium lactate group; SB, sodium bicarbonate group; NC, NaCl 0.9% group; MAP, mean arterial pressure; HR, heart rate; BP, blood pressure; PBP, pulmonary blood pressure; PCWP, pulmonary capillary wedge pressure; RAP, right atrial pressure; SvO2, mixed venous oxygen saturation; CI, cardiac index; SDF, sidestream dark field; NIRS, near-infrared spectroscopy; A-VBG, arterial and venous blood gas. (PDF 48 kb)

Additional file 3: Changes in right atrial pressure (RAP), systemic vascular resistance (SVR), systolic index (SI), and oxygen extraction ratio (OER). Open circles and dotted line: NC group (*n* = 5); squares and grey line: SB group (*n* = 5); closed circles and black line: SL group (*n* = 5). Results are expressed as median with interquartile ranges. *p < 0.05, NC vs SL; ^#^ *p* < 0.05, SB vs SL; &*p* < 0.05, NC vs SB. (PDF 174 kb)

Additional file 4: Changes in rest flow (RF) and peak flow (PF). Open circles and dotted line: NC group (*n* = 5); squares and grey line: SB group (*n* = 5); closed circles and black line: SL group (*n* = 5). Results are expressed as median with interquartile ranges. *p < 0.05, NC vs SL; ^#^ *p* < 0.05, SB vs SL; &*p* < 0.05, NC vs SB. (PDF 126 kb)

Additional file 5: Changes in pH, blood osmolality, bicarbonate, and sodium levels. Open circles and dotted line: NC group (*n* = 5); squares and grey line: SB group (*n* = 5); closed circles and black line: SL group (*n* = 5). Results are expressed as median with interquartile ranges. *p < 0.05, NC vs SL; ^#^ *p* < 0.05, SB vs SL; &*p* < 0.05, NC vs SB. (PDF 255 kb)

Additional file 6: Evolution of blood and urine biological parameters in the three groups. Results are expressed as median with interquartile ranges. Kruskal-Wallis test with Dunn’s multiple comparisons test and Mann-Whitney *U* test were used for intergroup comparisons. (PDF 47 kb)

## Abbreviations

CI: Cardiac index
DO_2_: Oxygen delivery
LPS: Lipopolysaccharide
MAP: Mean arterial pressure
MFI: Microvascular flow index
NC: 0.9% NaCl group
NIRS: Near-infrared spectrometer
OER: Oxygen extraction ratio
PaO_2_/FiO_2_: Arterial oxygen partial pressure and inspired oxygen fraction ratio
PF: Peak flow
Pv-aCO_2_: Venoarterial CO_2_ tension difference
RF: Rest flow
SB: Sodium bicarbonate group
SL: Sodium lactate group
StO_2_: Tissue oxygen saturation
SvO_2_: Mixed venous oxygen saturation
SVR: Systemic vascular resistance
VO_2_: Oxygen consumption
