# Supplementary figures and images for: Comparison of fluid balance and hemodynamic and metabolic effects of sodium lactate versus sodium bicarbonate versus 0.9% NaCl in porcine endotoxic shock: a randomized, open-label, controlled study
Source: Crit Care. 2017 May 19;21:113. doi: 10.1186/s13054-017-1694-1 (PMC5438514; doi:10.1186/s13054-017-1694-1)

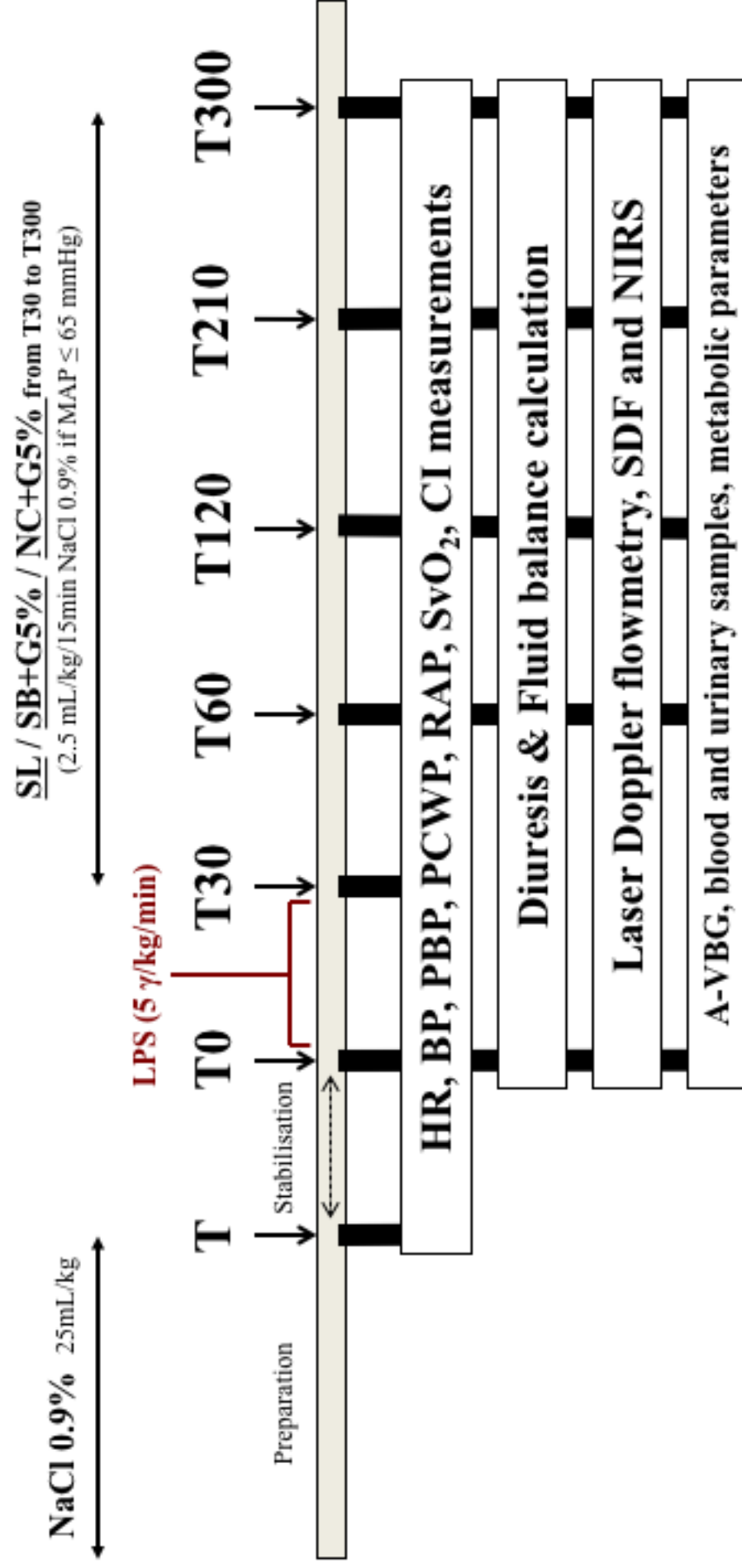

Supplement: Supplementary file 2 — Study design. During preparation period, all animals received 25 mL/kg 0.9% NaCl to prevent hypovolemia. When all preparations were completed, a 30-min period was allowed to stabilize the measured variables. Measurements were taken over a 5-h period. All animals were administered 5 μg/kg/min Escherichia coli lipopolysaccharide (LPS) (serotype 055:B5; Sigma Chemical Co., St. Louis, MO, USA). If MAP fell below 65 mmHg, 2.5 mL/kg infusion of NaCl 0.9% was given as rescue therapy every 15 min. We studied three groups receiving 450 mL (from T30 to T300) of different fluids as follows: 11.2% hypertonic sodium lactate AP-HP® (AGEPS, Paris, France) (SL group), 0.9% NaCl (NC group), and 8.4% hypertonic sodium bicarbonate (SB group). In order to inject an equivalent energy supply, 5% glucose solution (Baxter SAS, Guyancourt, France) was perfused in the NC and SB groups. Finally, in order to maintain the same fluid intake in the three groups, the SL group received 780 mL sterile water for injection (Baxter SAS, Guyancourt, France) in place of 5% glucose solution from T30 to T300. SL, Sodium lactate group; SB, sodium bicarbonate group; NC, NaCl 0.9% group; MAP, mean arterial pressure; HR, heart rate; BP, blood pressure; PBP, pulmonary blood pressure; PCWP, pulmonary capillary wedge pressure; RAP, right atrial pressure; SvO2, mixed venous oxygen saturation; CI, cardiac index; SDF, sidestream dark field; NIRS, near-infrared spectroscopy; A-VBG, arterial and venous blood gas. (PDF 48 kb) [file 13054_2017_1694_MOESM2_ESM.pdf]

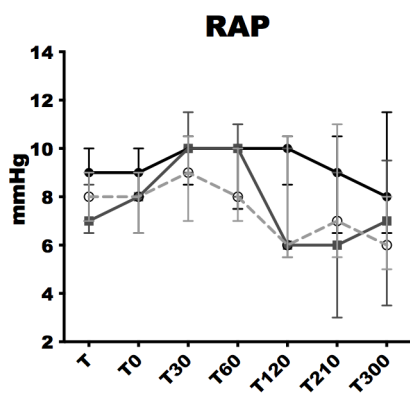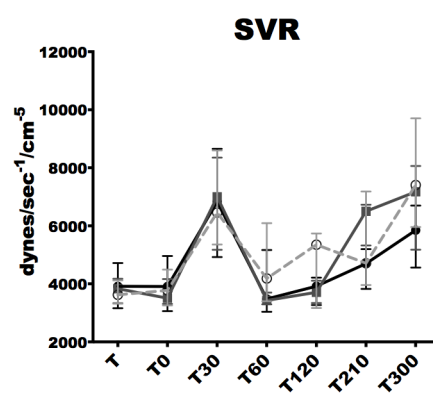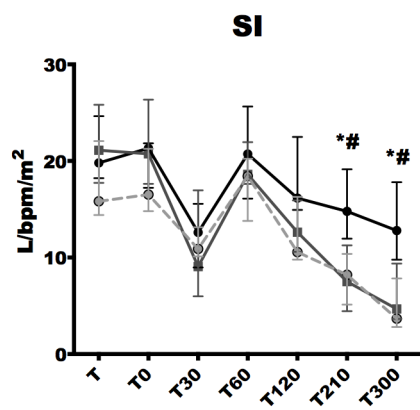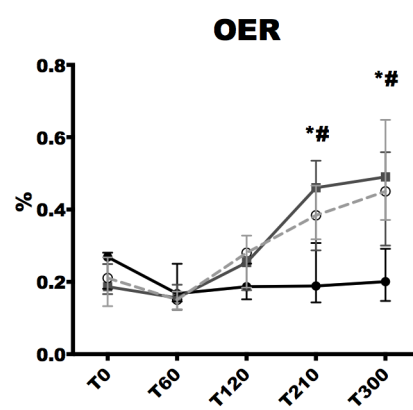

Supplement: Supplementary file 3 — Changes in right atrial pressure (RAP), systemic vascular resistance (SVR), systolic index (SI), and oxygen extraction ratio (OER). Open circles and dotted line: NC group (n = 5); squares and grey line: SB group (n = 5); closed circles and black line: SL group (n = 5). Results are expressed as median with interquartile ranges. *p < 0.05, NC vs SL; # p < 0.05, SB vs SL; &p < 0.05, NC vs SB. (PDF 174 kb) [file 13054_2017_1694_MOESM3_ESM.pdf]

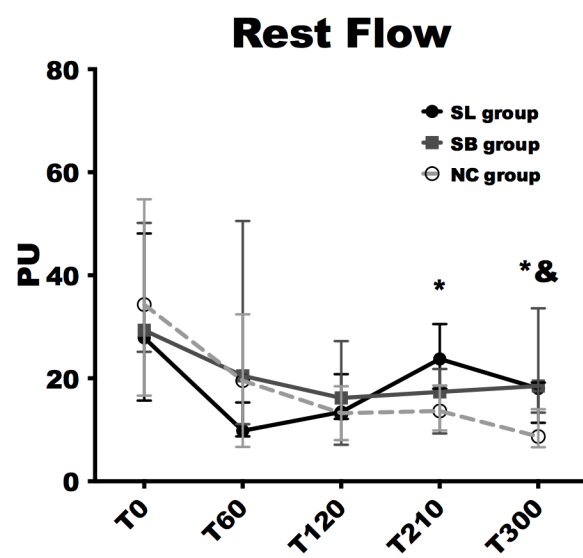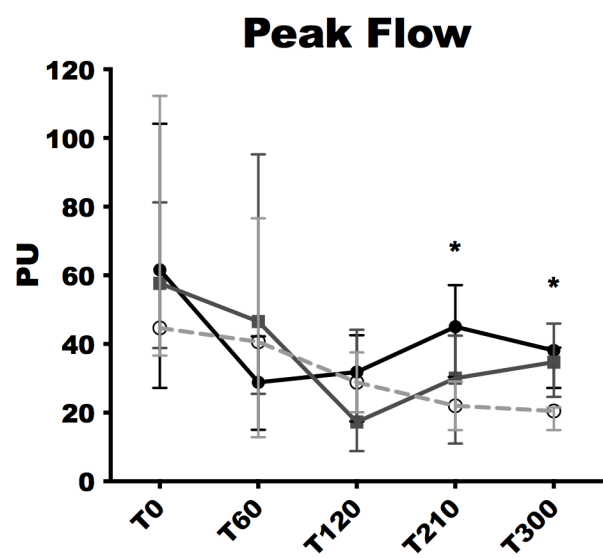

Supplement: Supplementary file 4 — Changes in rest flow (RF) and peak flow (PF). Open circles and dotted line: NC group (n = 5); squares and grey line: SB group (n = 5); closed circles and black line: SL group (n = 5). Results are expressed as median with interquartile ranges. *p < 0.05, NC vs SL; # p < 0.05, SB vs SL; &p < 0.05, NC vs SB. (PDF 126 kb) [file 13054_2017_1694_MOESM4_ESM.pdf]

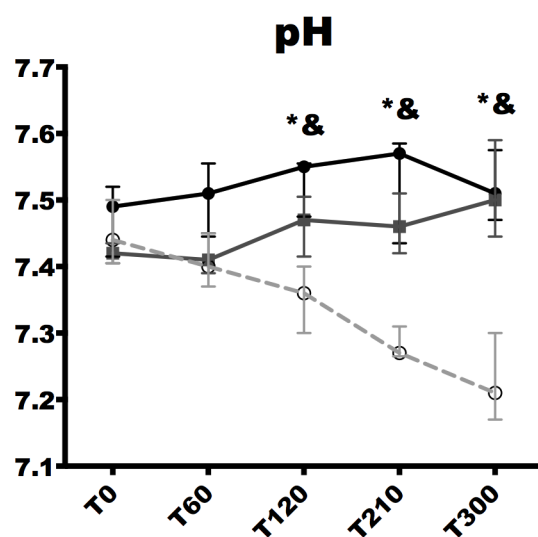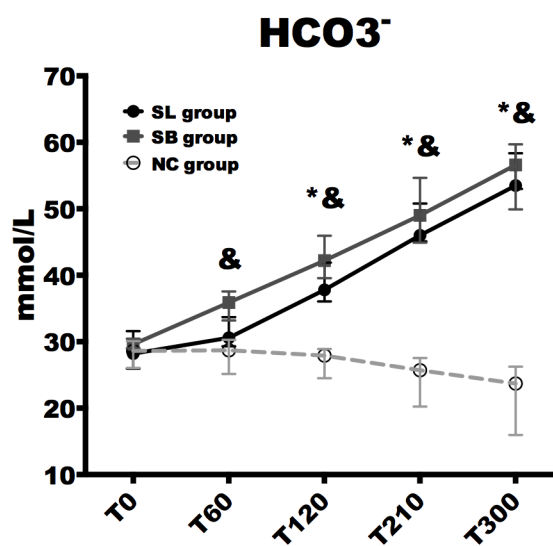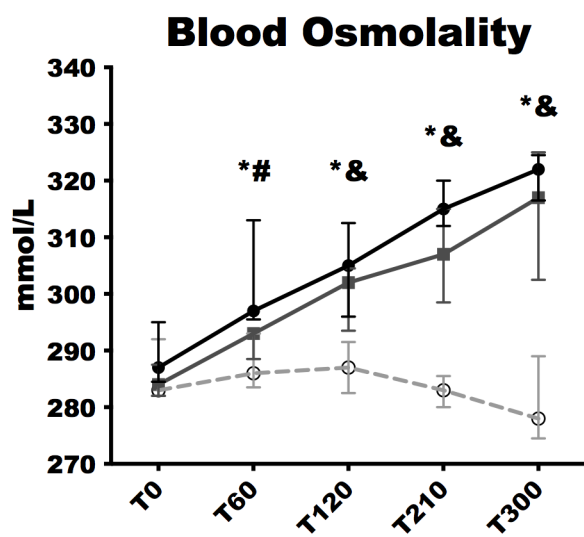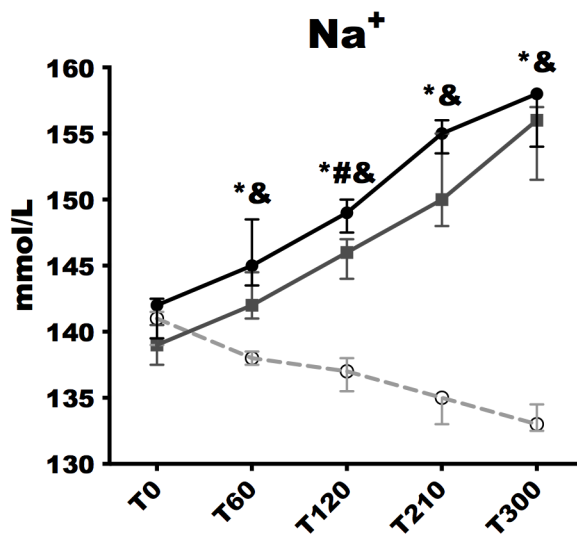

Supplement: Supplementary file 5 — Changes in pH, blood osmolality, bicarbonate, and sodium levels. Open circles and dotted line: NC group (n = 5); squares and grey line: SB group (n = 5); closed circles and black line: SL group (n = 5). Results are expressed as median with interquartile ranges. *p < 0.05, NC vs SL; # p < 0.05, SB vs SL; &p < 0.05, NC vs SB. (PDF 255 kb) [file 13054_2017_1694_MOESM5_ESM.pdf]
