# Supplementary material for: Comparison of fluid balance and hemodynamic and metabolic effects of sodium lactate versus sodium bicarbonate versus 0.9% NaCl in porcine endotoxic shock: a randomized, open-label, controlled study
Source: Crit Care. 2017 May 19;21:113. doi: 10.1186/s13054-017-1694-1 (PMC5438514; doi:10.1186/s13054-017-1694-1)
Supplement: Supplementary file 6 — Evolution of blood and urine biological parameters in the three groups. Results are expressed as median with interquartile ranges. Kruskal-Wallis test with Dunn’s multiple comparisons test and Mann-Whitney U test were used for intergroup comparisons. (PDF 47 kb) [file 13054_2017_1694_MOESM6_ESM.pdf]

		NC Group	SB Group	SL Group	p NC vs. SB	p NC vs. SL	p SB vs. SL
BLOOD							
pH	T0	**7.44 [7.4-7.5]**	**7.42 [7.4-7.3]**	**7.49 [7.41-7.52]**	**ns**	**ns**	**ns**
	T210	**7.27 [7.26-7.41]**	**7.46 [7.42-7.51]**	**7.57 [7.43-7.58]**	**0.008**	**0.008**	**ns**
	T300	**7.21 [7.17-7.3]**	**7.5 [7.44-7.59]**	**7.51 [7.47-7.57]**	**0.008**	**0.008**	**ns**
HCO3- (mmol/l)	T0	**28.6 [26-30]**	**29.6 [29-30.5]**	**28.2 [26-31.6]**	**ns**	**ns**	**ns**
	T210	**25.7 [20.2-27.5]**	**49 [45-54.6]**	**46 [45-50.8]**	**0.008**	**0.008**	**ns**
	T300	**23.7 [16-26.2]**	**56.6 [50-59.7]**	**53.5 [53-58.3]**	**0.008**	**0.008**	**ns**
PaCO2 (mmHg)	T0	**40.8 [35.2-47.9]**	**46.3 [43.4-48.8]**	**37.3 [33.3-47]**	**ns**	**ns**	**ns**
	T210	**56.1 [43.5-63.5]**	**67.10 [62.7-77.2]**	**49.4 [46.3-62.5]**	**0.05**	**ns**	**0.03**
	T300	**56.1 [38.7-71]**	**70 [58.9-75.5]**	**64.7 [56.4-74.7]**	**ns**	**ns**	**ns**
Na+ (mmol/l)	T0	**141 [139-141.5]**	**139 [137.5-140.5]**	**142 [139.5-142.5]**	**ns**	**ns**	**ns**
	T210	**135 [133-135]**	**150 [148-155]**	**149 [147.5-150]**	**0.008**	**0.008**	**ns**
	T300	**133 [132.5-134.5]**	**156 [151.5-157]**	**155 [154-158]**	**0.008**	**0.008**	**ns**
K+ (mmol/l)	T0	**3 [3-4]**	**3.9 [3.8-4]**	**3.8 [3.5-4.1]**	**ns**	**ns**	**ns**
	T300	**4 [3-4.5]**	**3,6 [3.4-4.5]**	**3,1 [2.9-3.6]**	**ns**	**ns**	**0.07**
Cl- (mmol/l)	T0	**104 [100.5-105.5]**	**101 [100-102.5]**	**103 [100-106.5]**	**ns**	**ns**	**ns**
	T300	**98 [95.5-104.5]**	**91 [90-91.5]**	**90 [89-94]**	**0.008**	**0.008**	**ns**
Ca2+ (mmol/l)	T0	**2.4 [2.3-2.5]**	**2.5 [2.4-2.6]**	**2.5 [2.1-2.6]**	**ns**	**ns**	**ns**
	T300	**2.3 [2.1-2.3]**	**1.98 [1.9-2.1]**	**2.2 [2.1-2.3]**	**0.03**	**ns**	**0.04**
Mg2+ (mmol/l)	T0	**17 [16.5-19]**	**17 [16.5-18]**	**16 [14.5-18.5]**	**ns**	**ns**	**ns**
	T300	**19 [17.5-22]**	**18 [16.5-20]**	**18 [13.5-18.5]**	**ns**	**ns**	**ns**
SID (mEq/l)	T0	**58.7 [57-64.6]**	**61 [60.2-61.7]**	**57.6 [56.2-64.8]**	**ns**	**ns**	**ns**
	T210	**55.4 [51.1-60.3]**	**79.2 [74.8-81]**	**72.3 [70.6-80.9]**	**0.008**	**0.008**	**ns**
	T300	**56 [50-62]**	**83.8 [79.2-85]**	**81.1 [77.7-85.9]**	**0.008**	**0.008**	**ns**
Total protein (g/l)	T0	**50 [48-56.5]**	**55 [53.5-59.5]**	**50 [48-55]**	**ns**	**ns**	**0.07**
	T210	**42 [35-47.5]**	**40 [40-44.5]**	**41 [41-46.5]**	**ns**	**ns**	**ns**
	T300	**43 [31.5-48.5]**	**41 [37.5-44.5]**	**43 [41.5-44**	**ns**	**ns**	**ns**
Osmolality (mosm/kg)	T0	**283 [282-292]**	**284 [282-287]**	**287 [284-295]**	**ns**	**ns**	**ns**
	T210	**283 [280-285]**	**307 [298-315]**	**315 [312-320]**	**0.008**	**0.008**	**ns**
	T300	**278 [274-289]**	**317 [302-325]**	**322 [316-324]**	**0.008**	**0.008**	**ns**
URINE							
Natriuresis (mmol)	T0	**15.2 [9.8-24.8]**	**15.7 [11.2-28.1]**	**5.3 [1.7-29.6]**	**ns**	**ns**	**ns**
	T210	**4.2 [1-6.4]**	**5 [3.7-14.3]**	**39.2 [18.5-43.7]**	**ns**	**0.008**	**0.01**
	T300	**0.3 [0.1-8.3]**	**4.5 [0.2-17.9]**	**24.7 [19.9-46.3]**	**ns**	**0.008**	**0.01**
Na/K	T0	**4.8 [3-6.3]**	**9.5 [7.6-21.3]**	**4.5 [3.5-8.7]**	**0.008**	**ns**	**0.09**
	T210	**4 [3.2-7.8]**	**7.8 [4.6-8.8]**	**13.5 [9.7-17.4]**	**ns**	**0.01**	**0.05**
	T300	**4.8 [3.4-6.7]**	**6.2 [3.2-11.1]**	**16.3 [9.9-18.8]**	**ns**	**0.01**	**0.03**
Chloruresis (mmol)	T0	**19.5 [12-26.3]**	**16.6 [11.4-25.2]**	**5.5 [0.8-28.3]**	**ns**	**ns**	**ns**
	T210	**5 [1.6-8.6]**	**3.8 [1.9-10.1]**	**15.7 [5.1-17.5]**	**ns**	**0.09**	**0.09**
	T300	**0.5 [0.2-9.9]**	**2.4 [0.09-4.7]**	**7.6 [3-11.3]**	**ns**	**0.15**	**0.09**
Osmolality (mosm/kg)	T0	**408 [345-543]**	**389 [324-519]**	**362 [179-649]**	**ns**	**ns**	**ns**
	T300	**369 [354-424]**	**335 [331-376]**	**350 [334-387]**	**ns**	**ns**	**ns**
